# Influence of Copper Oxide Nanoparticles on Gene Expression of Birch Clones In Vitro under Stress Caused by Phytopathogens

**DOI:** 10.3390/nano12050864

**Published:** 2022-03-04

**Authors:** Tatiana A. Grodetskaya, Peter M. Evlakov, Olga A. Fedorova, Vyacheslav I. Mikhin, Olga V. Zakharova, Evgeny A. Kolesnikov, Nadezhda A. Evtushenko, Alexander A. Gusev

**Affiliations:** 1 Research Institute of Innovative Technologies of the Forestry Complex, Laboratory of PCR Analysis, Voronezh State University of Forestry and Technologies Named after G. F. Morozov, 394087 Voronezh, Russia; tatyana.pokusina@yandex.ru (T.A.G.); fed-olga78@mail.ru (O.A.F.); dr.mikhin2018@yandex.ru (V.I.M.); nadya.evtushenko.94@mail.ru (N.A.E.); nanosecurity@mail.ru (A.A.G.); 2 Institute for Environmental Science and Biotechnology, Derzhavin Tambov State University, 392020 Tambov, Russia; olgazakharova1@mail.ru; 3 Department of Functional Nanosystems and High-Temperature Materials, National University of Science and Technology “MISIS”, 119991 Moscow, Russia; kea.misis@gmail.com; 4 Engineering Center, Plekhanov Russian University of Economics, 117997 Moscow, Russia

**Keywords:** CuO nanoparticles, birch, clonal micropropagation, phytopathogens, *Fusarium oxysporum*, *Fusarium avenaceum*, *Alternaria alternata*, sterilization, stress adaptation, gene expression

## Abstract

Recently, metal oxide nanoparticles (NPs) have attracted attention as promising components for the protection and stimulation of plant microclones in tissue culture in vitro. However, the effect of NPs on the genetic mechanisms underlying plant adaptive responses remains poorly understood. We studied the effect of column-shaped CuO NPs 50 nm in diameter and 70–100 nm in length at a concentration of 0.1–10 mg/L on the development of phytopathogenic fungi *Alternaria alternata*, *Fusarium oxysporum*, and *Fusarium avenaceum* in culture, as well as on the infection of downy birch micro-clones with phytopathogens and the level of genes expression associated with the formation of plant responses to stress induced by microorganisms. CuO NPs effectively suppressed the development of colonies of phytopathogenic fungi *A. alternata* and *F. avenaceum* (up to 68.42% inhibition at 10 mg/L CuO NPs) but not the development of a colony of *F. oxysporum*. Exposure to the NPs caused multidirectional responses at the level of plant genes transcription: 5 mg/L CuO NPs significantly increased the expression level of the *LEA8* and *MYB46* genes and decreased the expression of *DREB2* and *PAL*. Infection with *A. alternata* significantly increased the level of *MYB46*, *LEA8*, *PAL*, *PR-1*, and *PR-10* transcripts in birch micro-clones; however, upon exposure to a medium with NPs and simultaneous exposure to a phytopathogen, the expression of the *MYB46*, *PR-1*, and *PR-10* genes decreased by 5.4 times, which is associated with a decrease in the pathogenic load caused by the effect of NPs and the simultaneous stimulation of clones in vitro. The results obtained can be used in the development of preparations based on copper oxide NPs for disinfection and stimulation of plant phytoimmunity during clonal micropropagation of tree crops.

## 1. Introduction

Obtaining planting material for woody plants by clonal micropropagation is a modern intensive method of mass asexual reproduction in tissue and cell culture, in which the resulting plants are genetically identical to the original specimen. Since the culture media provide ideal conditions for the growth of microorganisms, plant tissue cultures must be created and maintained under aseptic conditions. Antibiotics are increasingly being used to reduce the risk of contamination in plants propagated in vitro. However, along with a bactericidal effect, antibiotics can have a toxic effect on plant tissues and inhibit the growth and development of explants [1,2]. In addition, it is known that microorganisms can adapt to biocidal drugs by mutations, which leads to the resistance of phytopathogens [3,4]. A promising alternative can be the use of nanoparticles (NPs) as sterilizing agents [5,6,7,8,9,10,11].

Copper-based NPs are particularly promising as antimicrobial agents against a wide spectrum of pathogens [12,13,14]. As plant protection agents, Cu-based NPs showed greater fungicidal efficacy than a commercial preparation based on Cu(OH)_2_ and ionic forms of copper [15].

It is known that copper-based NPs can have a positive effect on plant development both during traditional seed germination [16] and in tissue culture [17,18]. NPs are used as elicitors for the synthesis of bioactive compounds, since they can affect the secondary metabolism in plants, as well as in culture systems [19]. Metal NPs can reduce stress by increasing photosynthetic activity [20], the formation of phenolates, which can act as antioxidants in cases of excessive production of ROS [21] and by the up-regulation of stress-response genes encoding pathogenesis-related (PR) proteins and antioxidant enzymes [22], transcription factors (TFs) [23], associated with water stress late embryogenesis abundant (LEA) proteins, dehydrins, and aquaporins [24]. Iron, copper, cobalt, and zinc oxide nanoparticles improved relative water content, drought tolerance index, biomass reduction rate, and positively regulated resistance gene expression in drought-exposed soybean leaves and roots [25]. The combined use of elevated CO_2_- and Ni-based NPs improved growth and photosynthesis and mitigated nickel-induced oxidative stress in wheat [26].

However, there are still not enough works comprehensively explaining the genetic mechanisms of plant biosynthesis regulation in connection with the effect of NPs [27], although such studies were carried out on, e.g., pumpkin [28], barley [29,30], alfalfa [31], watermelon [32], bean [33], Indian mustard [34], thale cress [35,36,37], rice [38,39], soybean [40], lettuce [41], and wheat [42].

Previously, we revealed the positive effects of 50 nm thick copper oxide nanoplates on poplar microshoots during in vitro cultivation. However, the mechanisms of the plant responses formation at the genes expression level in woody plants remained unclear [43].

The aim of the study was to assess the effects of copper oxide NPs on the growth of *Alternaria alternata*, *Fusarium oxysporum*, and *Fusarium avenaceum* colonies, as well as on the expression of stress resistance genes in birch clones in vitro upon invasion with phytopathogen.

## 2. Materials and Methods

### 2.1. Nanoparticles and Nanoparticles Suspensions

We used copper oxide NPs (CuO NPs) by Sigma-Aldrich (St. Louis, MO, USA). The sample was an ultrafine black powder with a manufacturer’s declared particle size of <50 nm and a surface area of 29 m^2^/g.

The morphology and size of particles before the experiment were determined by scanning electron microscopy (SEM) on a Tescan Vega 3 microscope (Czech Republic). Elemental analysis of the powder was performed by energy-dispersive X-ray spectroscopy (EDX) using a 10 mm^2^ SDD Detector, namely X-Act energy dispersive analyzer (Oxford Instruments, Abingdon, UK).

The colloidal solution of NPs was prepared on the basis of sterile distilled water (pH 7.1 ± 0.2), containing 1 g/L CuO NPs. The portions of nanopowder were weighed using an analytical balance (OHAUS Corporation, Parsippany, NJ, USA) and poured into a previously prepared dispersion medium and stirred with a glass rod. After stirring, the suspension was treated in an Elmasonic S30H ultrasonic bath (Elma, Singen, Germany) for 15 min (with a break to cool the dispersions every 5 min).

The measurements of particle/aggregate size distribution in the prepared suspensions were taken at the temperature of 25 °C using the dynamic light scattering technique by means of the Malvern ZetaSizer Nano device (Westborough, MA, USA). To assess the stability of the NPs colloidal system, the zeta potential of the solution was also analyzed on the ZetaSizer Nano device.

### 2.2. Determination of Antifungal Activity

To confirm the ability of CuO NPs to suppress the growth of epiphytic microflora on the birch explants, which prevents the successful introduction of plants into tissue culture, a study of their antimicrobial activity against the phytopathogenic fungi *F. oxysporum*, *F. avenaceum,* and *A. alternata* was tested. To determine the antifungal activity, CuO NPs at concentrations of 0.1, 1, 5, and 10 mg/L were added to Czapek agar medium (Sigma-Aldrich, St. Louis, MO, USA). For the analysis, a disc of active mycelial growth with a diameter of 0.5 cm was cut out from a 7-day fungi culture, which was placed on Czapek’s medium containing NPs. As a control, inoculations of fungal strains on the NPs-free nutrient medium were used. The seeded plates were then incubated at 28 °C for 7 days. After 3, 5, and 7 days of observation, the fungal colonies’ diameters were assessed. The experiment was carried out in triplicate.

The antifungal effect of CuO NPs was calculated using Formula (1):

Inhibition rate (%) = [(D − d)/D] × 100,(1)

- where D is the diameter of the fungus colony in the control variant;
- d is the diameter of the NPs-treated fungus colony.

### 2.3. Cultivation and Artificial Infection

The apical shoots of *Betula pubescens* Ehrh. hybrid 15-1 selected by PhD Isakov I. Yu., associate professor at Voronezh State University of Forestry and Technologies, were obtained from the Unified genetic selection complex in the Voronezh region. The apical and axillary meristems from the experimental plants were introduced into the tissue culture and clonally propagated using the traditional technology [44,45]. CuO NPs were added to the nutrient medium at concentrations of 0.1, 1, 5, and 10 mg/L. The plants were maintained in a Binder KBW 400 (E5.1) climatic chamber (Binder, Tuttlingen, Germany) with the following climatic conditions: a 16-h photoperiod at an illumination of 30 μm/m^−2^·s^−1^ and a temperature of 24 ± 2 °C. Artificial infection of the microclones was carried out according to the method presented earlier [43]. The fungus culture at a concentration of 2 × 10^6^ spores/mL was sprayed onto the birch clones and simultaneously introduced into the cultivation media with and without NPs. The control samples were not infected. The microclones were taken after 2 days of the experiment, fixed at −80 °C, and used for further research.

### 2.4. Evaluation of the Influence of A. alternata and CuO NPs on the Expression of Stress Resistance Genes in Regenerants of Downy Birch

In the work, the influence of *A. alternata*, CuO NPs, and the synergistic action of the fungus and NPs on the expression of stress-resistance genes in regenerants of downy birch was assessed.

#### 2.4.1. RNA Extraction and Evaluation

RNA was extracted by the CTAB method using a modified protocol proposed by Rubio-Piña [46]. The extracted RNA was analyzed by electrophoresis in 1% agarose gel stained with the SYBR Green I intercalation dye (Lumiprobe, Cockeysville, MD, USA); the result was visualized using the Infinity VX2 1126MX X-Press gel documentation system (Vilber Lourmat, Collégien, France).

The RNA concentration was determined using a Qubit 2.0 fluorometer (Thermo Fisher Scientific, Waltham, MA, USA) according to the manufacturer’s instructions.

#### 2.4.2. cDNA Synthesis and Primers Picking

cDNA synthesis was performed using a standard kit with MMLV-RH (Dia-M, Russia) using 0.5–1 µg of total RNA.

The primers for resistance genes [43,47,48,49,50] were selected based on sequences from the NCBI database using the Primer3 program (Table 1).

#### 2.4.3. PCR Analysis and Assessment of the Stress Genes Expression

After optimizing the conditions for annealing the primers, the PCR protocol was used: 3 min at 95 °C, then 45 cycles of the following stages: 30 s at 95 °C, 30 s at 60 °C, 30 s at 72 °C, and the final elongation at 72 °C for 2 min. The reaction was carried out using a standard set of reagents containing SYBR Green I dye (Evrogen, Moscow, Russia) in real time.

Analysis of the genes expression was performed by the ΔΔCt method using the LightCycler480 II v 1.5.1 software (Roche, Basel, Switzerland) [51]. The *GAPDH* gene was used as a reference.

### 2.5. Statistical Analysis

All experiments were carried out in three biological and analytical replicates. Statistical data processing was carried out using Microsoft Excel 2010 (Descriptive Statistics package) using one-way analysis of variance (ANOVA) at a 5% significance level.

## 3. Results

### 3.1. Nanoparticles Characterization

**Figure 1:**
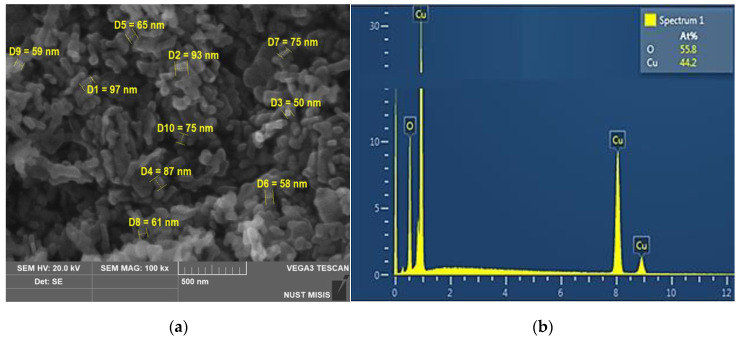
Electron microscopic examination of CuO NPs powder: (**a**) SEM image indicates that the studied material consists of nanoparticles; (**b**) EDX analysis confirms that the chemical composition of nanoparticles includes copper and oxygen.

SEM analysis revealed that the CuO NPs used in the work are rod-like structures with rounded ends assembled into aggregates (Figure 1a).

As can be seen from the presented micrograph, the average diameter of individual particles was about 50 nm, the length was 70–100 nm. Energy-dispersive X-ray spectroscopy showed the analyzed powder to be copper oxide, without any impurities (Figure 1b).

**Figure 2:**
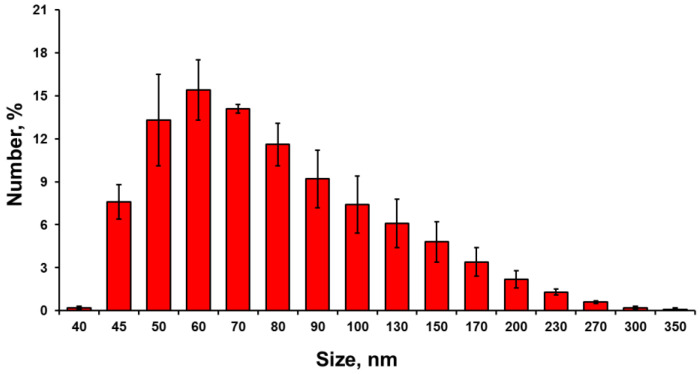
Dispersed composition of CuO NPs suspension (1 g/L) confirms that the particles in suspension have nanometer sizes.

During the analysis of the particle size in solution, it was found that the average size of NPs and their aggregates was 40–300 nm (Figure 2). Most of the particles were in the range of 50–80 nm.

**Figure 3:**
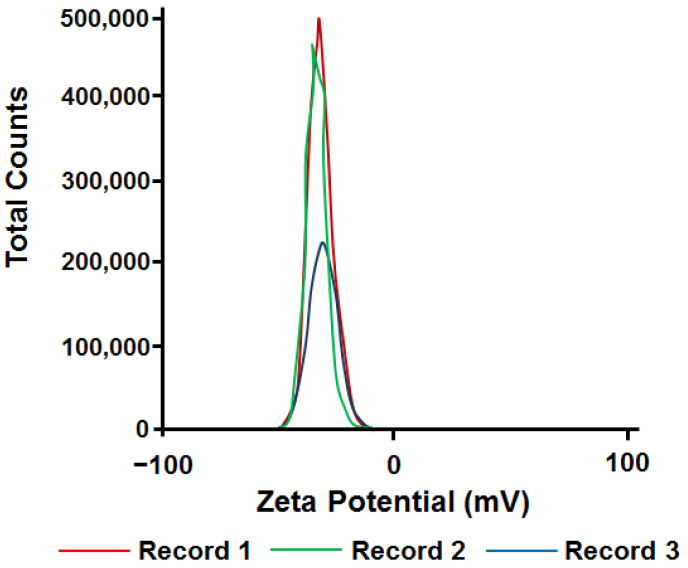
Zeta potential of CuO NPs in 1 g/L suspension.

The zeta potential measurement showed values of about 32 mV (Figure 3), which indicates a high stability of the system.

Thus, the results proved that the investigated particles were copper oxide NPs, without impurities. According to electron microscopy and dynamic light scattering technique, the average particle size was 50–100 nm.

### 3.2. Fungicidal Action of CuO NPs

Since fungus-induced diseases are more common than the ones caused by bacteria, we studied the antifungal activity of the tested NPs colloidal solutions against the most common plant pathogens.

**Figure 4:**
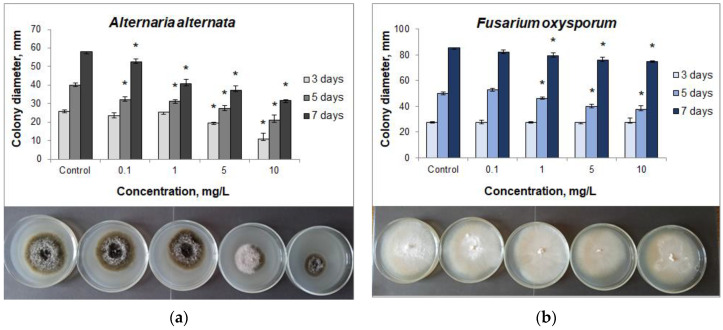

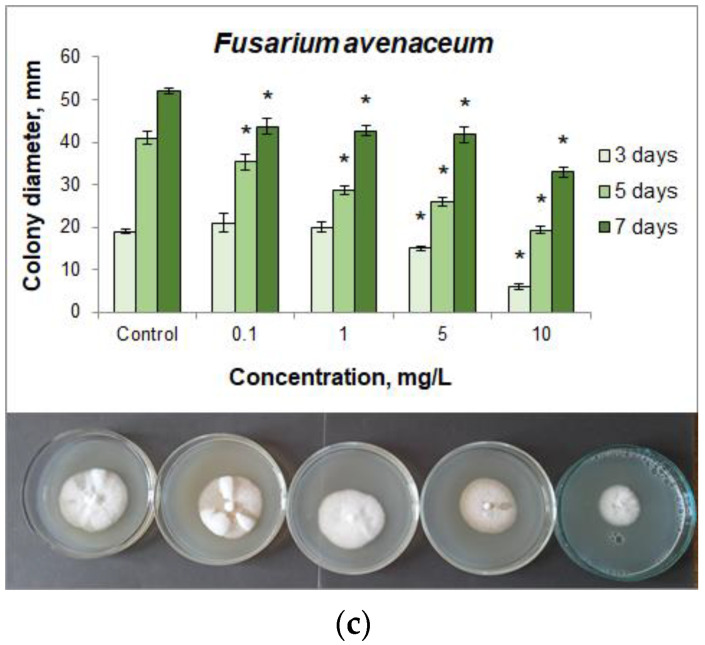
Influence of CuO NPs on the growth of phytopathogenic fungal colonies *Alternaria alternata* (**a**), *Fusarium oxysporum* (**b**), and *Fusariun avenaceum* (**c**) on days 3, 5, and 7 of growth under the influence of NPs (* is significant deviation from control). The difference in the size of a fungal colony was shown on Czapek’s medium containing various concentrations of nanoparticles on the 7th day of growth.

The results obtained showed that the highest antifungal activity of CuO NPs in relation to all the studied isolates in terms of the degree of phytopathogenic fungal colony growth suppression was observed when 10 mg/L NPs was added to the nutrient medium (Figure 4a–c).

It should be noted that high inhibition rates of the colony growth (up to 68%) were shown in relation to *A. alternata* and *F. avenaceum*. The level of *F. oxysporum* growth inhibition was insignificant (24%), which indicates the possible resistance mechanisms in this microorganism (Table 2).

Analysis showed the maximum inhibitory effect of NPs on the growth of the fungal colonies at the early stages of cultivation, namely, up to 3 days for *A. alternata* and *F. avenaceum*, and up to 5 days for *F. oxysporum*. On the 7th day of the experiment the inhibition rate decreased, which indicated the possible development of resistance in the microorganisms. Lower NPs concentrations (1 and 5 mg/L) showed the highest level of the colony growth inhibition on the 7th day of the experiment. The maximum inhibition level of *A. alternata* and *F. avenaceum* was recorded at concentrations of 1, 5, 10 mg/L, and 10 mg/L, respectively.

Thus, the revealed activity of CuO NPs against the growth of *A. alternata*, *F. oxysporum*, and *F. avenaceum* phytopathogenic fungal colonies makes it possible to use them as components to reduce the risk of contamination during clonal micropropagation of woody plants.

### 3.3. Influence of CuO NPs on Infected Birch Explants at the Stage of Introduction into Culture

**Figure 5:**
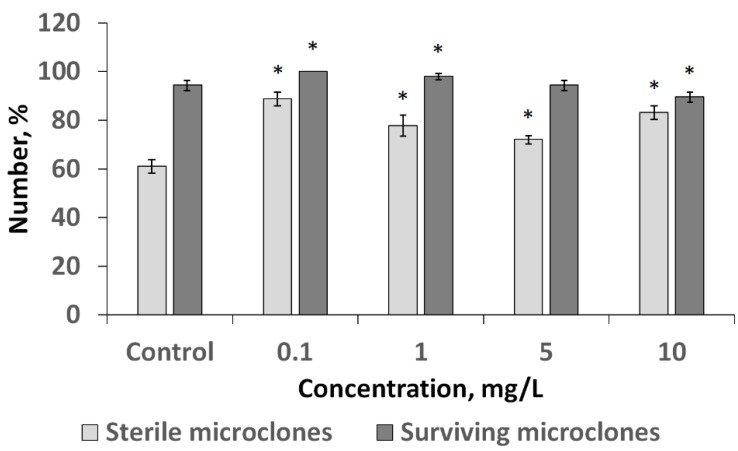
Influence of CuO NPs on explants of downy birch at the stage of introduction into culture. A significant increase in sterile microclones was shown using 0.1 mg/L NPs (* is significant deviation from control). With increasing concentration, this value decreased, but remained higher than the control.

The effect of CuO NPs as a sanitizing agent was investigated when birch was introduced into tissue culture (Figure 5).

As shown in Figure 5, the investigated NPs at every tested concentration increased the number of sterile explants. Thus, in the control variant where the NPs were not used, this indicator was 60.0%, while in the experimental variants it ranged from 71.7 to 86.7%. In this case, the maximum suppression of microorganisms on the surface of the explants was observed at the minimum concentration of 0.1 mg/L. Analysis of the viability of birch explants on the nutrient medium after 3 weeks of cultivation showed that the tested concentrations are 0.1; 1; 5 mg/L had no phytotoxic effect as the amount of viable explants was not lower than that in the control variant of the experiment. At CuO NPs concentrations of 0.1 and 1 mg/L, the number of viable explants was, by 5.3% and 3.5%, higher than in the control, which indicates their stimulating effect.

### 3.4. Influence of A. alternata and CuO NPs on the Expression of Stress Resistance Genes in Regenerants of Downy Birch

The effect of NPs on the induced resistance of woody plants to diseases was studied against an artificial infectious background created by the phytopathogenic fungus *A. alternata*. For this purpose, we analyzed the samples grown without NPs, infected with *A. alternata,* and exposed to 5 mg/L CuO NPs. The influence of CuO NPs without the phytopathogen was also investigated. *A. alternata* is one of the most dangerous phytopathogens as it causes necrosis of cuttings as well as lodging of shoots and seedlings [15]. In addition, it was experimentally found that that the influence of the NPs caused the most lasting effect in relation to this phytopathogen as after 7 days of observation the suppression of fungal growth was 45.3% (Table 2). The CuO NPs concentration of 5 mg/L was chosen for the study as it showed a high inhibition level of the phytopathogenic fungi while having no adverse effects upon the viability of explants when introduced into the tissue culture.

**Figure 6:**
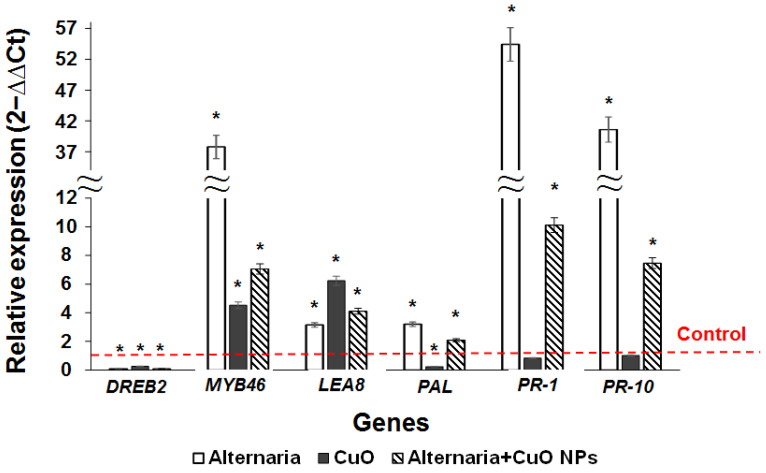
Expression of resistance genes in birch microclones. White bars—microclones infected with *A. alternata*, gray bars—plants cultivated on the medium containing 5 mg/L CuO NPs, striped bars—microclones infected with *A. alternata* and exposed to 5 mg/L CuO NPs, and control—plants on the medium without NPs and not exposed to the phytopathogen (* is significant deviation from control). The level of *MYB46*, *PAL*, *PR-1,* and *PR-10* expression was lower under combined exposure to NPs and *A. alternata* than under exposure to a single phytopathogen.

The study revealed significant activation of *MYB46*, *LEA8*, *PAL*, *PR-1,* and *PR-10* genes in the infected plants cultivated in the NPs free medium (Figure 6).

Exposure to CuO NPs at a concentration of 5 mg/L caused a significant increase in the level of *LEA8* and *MYB46* genes expression in the birch clones in vitro, while the expression of *DREB2* and *PAL* was reduced, and the level of *PR-1* and *PR-10* did not exceed the control variant. Under the combined influence of *A. alternata* and CuO NPs, the expression of *MYB46*, *LEA8*, *PAL*, *PR-1*, and *PR-10* genes was activated, while the level of *DREB2* gene transcripts was decreased (Figure 6). The observed increase in the expression of the analyzed genes upon exposure to the phytopathogen was significantly higher without exposure to NPs. At the same time, when cultivated on a medium containing NPs, the expression of *MYB46*, *PR-1*, and *PR-10* genes was significantly higher than the level in the control, but at the same time decreased by 5.4 times compared to the variant infected with *A. alternata* and incubated on the medium without CuO NPs. The data obtained show the stimulating effect of NPs on the expression of individual stress resistance genes in microclones. At the same time, the use of CuO NPs reduced the stress response level upon infection with *A. alternata*, which indicates their inhibitory effect on the growth of the pathogen.

## 4. Discussion

As we expected, CuO NPs had pronounced concentration-dependent antifungal effects on phytopathogens in culture. This is consistent with the results of a number of previous studies, where the authors demonstrated the suppression of the phytopathogenic fungi growth such as *Botrytis cinerea* [52], *F. oxysporum* [53], *Aspergillus* sp., *A. alternata* [54], *Fusicladium oleagineum*, and *Colletotrichum* sp. [55]. Both the diffusion of copper ions, which is an antimicrobial agent [56], and specific nanotoxic effects, such as the induction of oxidative stress [57] or damage to the cell membrane [58], are considered as possible mechanisms.

Interestingly, the maximum sterility of microplants was observed at the lowest of the studied NPs concentrations. It may be surmised that the effect is achieved not due to a direct destruction of phytopathogenic microorganisms by NPs, but indirectly, through the stimulation of phytoimmunity. NPs at low concentrations can cause moderate stress in plants, one of the reactions to which is a change in their biochemical status [59]. Compounds such as peroxidases and polyphenols begin to be produced, which are part of the nonspecific plant defense system against phytopathogenic microorganisms [60]. An increase in the concentration of NPs intensifies the “nano” induced stress [61], and the overall efficiency of plant adaptation to stress begins to decrease, which is ultimately manifested by a reduced number of viable microclones at the maximum concentration of NPs.

Since the biosynthesis of chemical agents of nonspecific phytoimmunity is controlled by certain genes, the revealed changes in the expression profile of plant genes under the influence of CuO NPs and the phytopathogenic fungus *A. alternata* can serve as a confirmation of this theory.

Exposure to NPs promoted the activation of *MYB46* and *LEA8* genes expression, which act as factors of nonspecific protection upon stress exposure. MYB46 transcription factor triggers a transcriptome response through the regulation of genes responsible for cell wall metabolism and cell matrix reorganization. According to [62], MYB46 acted as a modulator of susceptibility to infection with *B. cinerea* in *A. thaliana*, facilitating the integration of cell wall remodeling and subsequent activation of secondary lines of defense, including class III peroxidases. Since phytopathogens penetrate into plant cells damaging the plant cell wall, the reorganization of its components is a necessary mechanism of adaptation to stress. The effect of CuO NPs on birch microclones activates the protective proteins of osmoregulation, as evidenced by an increase in the expression of *LEA8* gene, which belongs to the class of dehydrins from the family of late embryogenesis proteins. LEA8 protein can provide protection against phytopathogens invasion by maintaining the integrity of cells and cellular structures, directly binding to their surface or ordering water around bound macromolecules [63]. Thus, the impact of NPs stimulates the development of nonspecific immunity required under the influence of stress, including infection with phytopathogens.

*A. alternata* contamination promoted the development of a stress response in birch microclones, as evidenced by an increase in gene expression of the key player from the phenylpropanoid pathway, phenylalanine ammonia lyase (PAL) [64], representatives of the protection proteins (LEA) [63], pathogenesis-related proteins (PR) [65] and transcription factors (MYB) [66]. Exposure to CuO NPs caused a decrease in the level of *PAL* gene transcripts, while a positive effect of CuO NPs on the expression of this gene in cabbage roots and seedlings [67], soybean roots [40], and cucumber shoots [68] was shown. Previous research revealed the activation of *DREB2* under the influence of *A. alternata* exposed to CuO and Ag NPs, as well as under the separate action of NPs on poplar microclones [43]; however, our results indicate a decrease in the expression of *DREB2* gene in birch microclones in all the experimental variants. The observed effects can be explained by the genotypic features of downy birch and the selected concentration of NPs, although a more detailed study of the NPs effect on the genes expression of woody plants is needed.

CuO NPs significantly downregulated the expression of *MYB46*, *PR-1*, and *PR-10* genes in comparison with infected plants not exposed to CuO NPs. The observed effect can be explained by the suppression of the phytopathogen activity by NPs, on the one hand, and their stimulating effect on birch microclones, on the other. Although the results obtained may indicate that CuO NPs contributed to a decrease in the negative effect of *A. alternata* on birch microclones, additional studies are required to endorse the sterilizing effect of CuO NPs and their contribution to boosting of a plant immunity.

We believe that our data confirm the promising nature of using copper oxide NPs to improve the technique of in vitro plant tissue culture. Future research should focus on identifying the mechanisms by which NPs affect plants and phytopathogens in order to create the best strategies for using NPs in biotechnology.

## Figures and Tables

**Table 1:** Primer sequences for the stress resistance genes.

Gene	Sequence (5′→3′)
*DREB2*	F: AGGCAGAGAACATGGGGAAA
	R: GAAAGTTGAGGCGAGCGTAA
*MYB46*	F: GATGTCGCTAGAAATGCCGG
	R: GTTGTCTGTACGCCCTGG
*LEA8*	F: AATGACTTTGACATGGGCGT
	F: AATGACTTTGACATGGGCGT
*PAL*	F: CTGTGGCTGCAACGGTTT
	R: TCAATTTGAGGTCCGAGCCA
*PR-1*	F: CCTCAAAGCCCACAATGACG
	R: TCTCGTCCACCCATAGCTTC
*PR-10*	F: GGCCCGGAACCATTAAGAAG
	R: CCACCCTCGATCAAGCTGTA
*GAPDH*	F: CAGCCGAAGATGTCAATGCA
	R: GGCCACTTGTTTGCTACCAA

**Table 2:** The level of fungal growth inhibition under the influence of CuO NPs solutions.

Pathogen	NPs Concentration, mg/L	Level of Inhibition (%)		
		3 Days	5 Days	7 Days
*Alternaria alternata*	0.1	10.39	19.25	9.14
	1	2.69	21.75	31.03
	5	24.23	31.75	35.69
	10	57.69	47.50	45.34
*Fusarium oxysporum*	0.1	1.44	0	3.52
	1	0	7.40	7.03
	5	1.44	20.00	10.81
	10	0	24.60	12.08
*Fusarium avenaceum*	0.1	10.53	13.90	15.96
	1	5.26	30.00	17.88
	5	21.05	36.59	19.81
	10	68.42	52.93	36.54
